# A comparative study of amplitude of low-frequence fluctuation of resting-state fMRI between the younger and older treatment-resistant depression in adults

**DOI:** 10.3389/fnins.2022.949698

**Published:** 2022-08-25

**Authors:** Jifei Sun, Chunlei Guo, Yue Ma, Zhongming Du, Zhi Wang, Yi Luo, Limei Chen, Deqiang Gao, Xiaojiao Li, Ke Xu, Yang Hong, Xue Yu, Xue Xiao, Jiliang Fang, Yong Liu

**Affiliations:** ^1^Guang’anmen Hospital, China Academy of Chinese Medical Sciences, Beijing, China; ^2^Dongzhimen Hospital, Beijing University of Chinese Medicine, Beijing, China; ^3^Beijing First Hospital of Integrated Chinese and Western Medicine, Beijing, China; ^4^Affiliated Hospital of Traditional Chinese Medicine, Southwest Medical University, Luzhou, China

**Keywords:** treatment-resistant depression (TRD), amplitude of low-frequency fluctuation, MRI, major depressive disorder (MDD), age

## Abstract

**Background:**

Treatment-resistant depression (TRD) may have different physiopathological neuromechanism in different age groups. This study used the amplitude of low frequency fluctuations (ALFF) to initially compare abnormalities in local functional brain activity in younger and older patients with TRD.

**Materials and methods:**

A total of 21 older TRD patients, 19 younger TRD, 19 older healthy controls (HCs), and 19 younger HCs underwent resting-state functional MRI scans, and the images were analyzed using the ALFF and further analyzed for correlation between abnormal brain regions and clinical symptoms in TRD patients of different age groups.

**Results:**

Compared with the older TRD, the younger TRD group had increased ALFF in the left middle frontal gyrus and decreased ALFF in the left caudate nucleus. Compared with the matched HC group, ALFF was increased in the right middle temporal gyrus and left pallidum in the older TRD group, whereas no significant differences were found in the younger TRD group. In addition, ALFF values in the left middle frontal gyrus in the younger TRD group and in the right middle temporal gyrus in the older TRD were both positively correlated with the 17-item Hamilton Rating Scale for Depression score.

**Conclusion:**

Different neuropathological mechanisms may exist in TRD patients of different ages, especially in the left middle frontal gyrus and left caudate nucleus. This study is beneficial in providing potential key targets for the clinical management of TRD patients of different ages.

## Introduction

Major depressive disorder (MDD) is a common clinical psychiatric disorder with depressed mood, decreased cognitive function and somatic disorders as the main clinical features (Lawson et al., 2017). Epidemiological surveys show that MDD is expected to be the number one disease burden globally by 2030 (Ho et al., 2018). In addition, MDD is the leading cause of disability worldwide, with approximately 800,000 deaths by suicide each year (Fabbri et al., 2021). However, despite numerous studies, 30–40% of MDD patients still do not respond significantly antidepressants (Bergfeld et al., 2018). This type of MDD that does not respond significantly to two adequate doses and courses of antidepressant medication can be called treatment-resistant depression (TRD) (Gaynes et al., 2020). TRD is a complex subtype of MDD with lower quality of life, higher costs and more severe activity impairment than non-TRD (Qiao et al., 2017; Jaffe et al., 2019). Therefore, understanding the pathogenesis of TRD and exploring potential biomarkers are of great importance to guide clinical treatment.

Patients with TRD may have differences in clinical symptoms at different ages. Previous studies have shown that younger TRD have more severe depressive symptoms than older TRD (Conelea et al., 2017). In addition, older TRD tend to have more severe cognitive and somatic dysfunction (Mulsant and Pollock, 1998; Knöchel et al., 2015). It has also been shown that age has been shown to be a moderator of response to treatment with numerous antidepressants (Ochs-Ross et al., 2020). Therefore, these different clinical symptoms suggest that different neuropathological mechanisms may exist in TRD patients of different ages.

However, there are fewer studies on the age classification of younger and older TRD patients, and there is no uniform consensus. A study defined the age of younger TRD patients as under 60 years (<60 years), while older TRD patients were defined as over 60 years (≥60 years), suggest that the younger group was more likely to have a history of psychiatric hospitalization and higher depression severity scores (Conelea et al., 2017). Another study defined the age range of TRD in adolescents as 14–17 years (Ghaziuddin et al., 2011). In addition, some studies have defined the age range of older TRD as 55–72 years (Lijffijt et al., 2022) and 65–84 years (Gronemann et al., 2020), but these studies were single-age studies and lacked the effect of different age boundaries on the primary outcome. Therefore, the neurobiological evidence for the age boundary between younger and older TRD is unclear and further clinical studies are necessary to elucidate it.

In recent years, resting state-functional functional magnetic resonance (rs-fMRI) has been gradually applied in the field of psychiatric disorders, including MDD (Wang et al., 2020; Liu P. et al., 2021), autism (Guo et al., 2017), and bipolar disorder (Zhang et al., 2021a). Amplitude of low-frequency fluctuation (ALFF) is a commonly studied metric in rs-fMRI and is able to describe the intensity of spontaneous brain activity in the resting state from an energy perspective (Zang et al., 2007). In addition, ALFF has been recently applied to clinical studies of MDD subtypes of disease (Guo et al., 2012; Liu et al., 2015). Only one study of ALFF at different ages in MDD with a first episode and without medication, and the abnormal brain regions in both groups were concentrated in the frontal, temporal, parietal, and occipital lobes (Guo et al., 2013). Up to date, little is known about the neuroimaging differences between younger and older TRD.

However, the use of an earlier age of onset to differentiate between different age groups of MDD patients is susceptible to severe psychopathology and risk factors (Klein et al., 1999; Zisook et al., 2007). Previous studies have found that age-related changes in affective, cognitive, and reasoning functions stabilize between the ages of 20 and 60 (Hedden and Gabrieli, 2004). In addition, another clinical study with a large sample observed different clinical symptoms of MDD in early onset depression (EOD) and late onset depression (LOD), using a cut-off age of 40 years (Korten et al., 2012). Therefore, we focused on younger TRD (21–40 years) and older TRD (41–60 years) as the age division range. This study was based on ALFF and focused on the differences in local functional brain activity between younger TRD and older TRD patients. In addition, to explore whether there is a correlation between abnormal brain area alters and clinical symptoms in the TRD group. This study will provide some insight into understanding the neuropathological mechanisms of TRD at different ages.

## Materials and methods

### Participants

A total of 40 outpatients with TRD from Guang’anmen Hospital, China Academy of Chinese Medical Sciences, Beijing First Hospital of Integrated Chinese and Western Medicine, and Xuanwu Hospital of the Capital Medical University, were recruited for this study. All patients with TRD showed the initial diagnosis of MDD in the fifth edition of the American Diagnostic and Statistical Manual of Mental Disorders (DSM-V). The inclusion criteria were as follows: (1) age, 21–60 years; (2) 17-item Hamilton depression rating scale (HAMD-17) score > 17; (3) right-handedness; (4) no response to two or more adequate doses and courses of antidepressant therapy. Thirty-eight gender- and age-matched healthy controls (HCs) (16 men and 22 women) were included in the HC group, which reflected the following: (1) age, 21–60 years; (2) HAMD-17 score < 7; (3) right-handedness; (4) no history of any mental illness in first-degree relatives.

The exclusion criteria for patients and HCs were as follows: (1) serious mental illness and other diseases such as cardiovascular and cerebrovascular disorders; (2) history of drug and alcohol abuse; (3) any contraindications to MRI, such as presence of a heart pacemaker, metal fixed false teeth, or severe claustrophobia; (4) pregnant or lactating status; (5) bipolar disorder or suicidal ideation.

All patients were required to sign an informed consent form before enrollment. This study was approved by the ethics committee of Guang’anmen Hospital, China Academy of Chinese Medical Sciences.

### Clinical materials and subgroups

In this study, we collected clinical information on all participants, including gender, age, years of education, and duration of illness. Patients in the TRD group were diagnosed by experienced psychiatrists and assessed for depression severity using the HAMD-17 scale. According to previous studies (Korten et al., 2012), all patients were divided into younger TRD group (21–40 years) and older TRD group (41–60 years). The HC group was also divided into two subgroups: younger HC group (21–40 years) and older HC group (41–60 years).

### Scan acquisition

All subjects in this study underwent MRI using a Magnetom Skyra 3.0 T scanner (Siemens, Erlangen, Germany), and the scans were performed at Guang’anmen Hospital, China Academy of Chinese Medical Sciences, and the scan parameters were the same. Before the scanning procedure, the subjects were instructed to remain awake and avoid active thinking. During the scanning process, the subjects were required to wear earplugs and noise-canceling headphones, to use a hood to immobilize the head, and to lie flat on the examination bed. The scanning procedure involved a localizer scan, high-resolution three-dimensional T1-weighted imaging, and BOLD-fMRI.

The scanning parameters were as follows: for three-dimensional T1-weighted imaging, time repetition/time echo = 2500/2.98 ms, flip angle = 7°, matrix = 64 × 64, field of view = 256 mm × 256 mm, slice thickness = 1 mm, slice number = 48, slices = 192, scanning time = 6 min 3 s; for BOLD-fMRI, time repetition/time echo = 2000/30 ms, flip angle = 90°, matrix = 64 × 64, field of view = 240 mm × 240 mm, slice number = 43, slice thickness/spacing = 3.0/1.0 mm, number of obtained volumes = 200, and scanning time = 6 min 40 s.

### Image processing

#### fMRI data preprocessing

The acquired rs-fMRI data were preprocessed using MATLAB-based DPARSF 5.1 software (DPARSF 5.1^1^) (Chao-Gan and Yu-Feng, 2010), as follows: (1) conversion of DICOM raw data to NIFTI format; (2) removal of the first 10 time points to stabilize the data; (3) slice timing; (4) realignment of head motion (removal of patients with head movements greater than 2 mm in any direction and motor rotation greater than 2°); (5) the resulting aligned image time series for each subject were each co-registered with the corresponding 3D T1-weighted image and the Diffeomorphic Anatomical Registration Through Exponentiated Lie Algebra (DARTEL) tool was used to normalize the data for all subjects to Montreal Neurological Institute (MNI) space, which was performed using the MNI coordinate space with 3mm × 3mm × 3mm; (6) linear detrending in order to reduce the influence of MRI equipment; (7) regression of covariates, including brain white matter signal, cerebrospinal fluid signal, and head movement parameters; (8) smoothening (a 6-mm full-width at half-maximum Gaussian kernel).

#### Amplitude of low frequency fluctuations analysis

Data were spatially normalized and smoothed, and a fast Fourier transform was performed to switch the time series to the frequency domain to obtain the power spectrum. The square root of the power spectrum at each frequency was calculated to obtain the average square root of the ALFF measurement for each voxel in the range of 0.01–0.08 Hz. Finally, time bandpass filtering (0.01–0.08 Hz) was performed. To reduce the inter-individual variability, ALFF was transformed to zALFF using Fisher’s *z* transformation before statistical analysis.

### Statistical analyses

#### Clinical data analysis

Clinical data were analyzed using the SPSS 23.0 statistical software (IBM Corporation, Somers, NY, United States). One-way analysis of variance was used to compare age and educational level among the four groups, and the chi-square test was used to compare gender differences. A two-sample *t*-test was used to compare the duration of disease and HAMD-17 scores between the two patient groups, with a threshold of *P* < 0.05 (two-tailed) set as statistically significant.

#### fMRI data analysis

##### Within-group patterns

Imaging data were analyzed using the DPARSF toolbox, and a voxel-based one-way analysis of variance was performed to compare the whole-brain ALFF map among the four groups. Gender, age, years of education, and framewise displacement (a metric derived from Jenkinson’s formula < 0.2) were used as covariates, and brain areas with ALFF differences among the four groups were corrected for Gaussian random fields (GRF). The corrected cluster level was set at *P* < 0.05 (two-tailed), and threshold voxel levels of *P* < 0.005 were defined as statistically different. The threshold was set to clusters > 20 voxels.

##### Between-group differences

We extracted the mean ALFF values of abnormal brain regions in each of the four groups and performed *post hoc* between-group 2-sample *t*-test analysis in SPSS 23.0 software to show the difference between each two groups (younger TRD group vs. older TRD group, younger TRD group vs. younger HC group, older TRD group vs. older HC group, younger HC group vs. older HC group). And using Bonferroni correction, the threshold was statistically significant at *P* < 0.0125 (0.05/4).

##### Correlations with symptoms

To verify the relationship between clinical symptoms and abnormal brain areas in the younger TRD group and the older TRD group, we performed Pearson correlation analysis between ALFF values and HAMD-17 scores for abnormal brain areas extracted from the two groups separately. Significance was set at a statistical threshold of *P* < 0.05 (two-tailed).

## Results

### Characteristics of research datasets

Two older TRD patients were excluded because of excessive head movement displacement. Therefore, a total of 19 younger TRD patients, 19 older TRD patients, 19 younger HCs, and 19 older HCs met the inclusion criteria. There were no significant differences between the younger TRD group and the older TRD group in terms of gender, years of education, duration of illness, and HAMD-17 scores. There were no statistical differences between the younger TRD group and the older TRD group in terms of gender, age, and years of education when compared to matched controls in each age group (Table 1).

**TABLE 1:** Demographic and clinical characteristics of the study participants.

Variable	Younger TRD (*n* = 19)	Older TRD (*n* = 19)	Younger HCs (*n* = 19)	Older HCs (*n* = 19)	*t(F)/*χ 2	*P*-value
Gender (M/F)	9/10	7/12	9/10	7/12	0.864	0.834^a^
Age (years)	33.10 ± 5.38	50.42 ± 5.98	31.36 ± 4.24	50.63 ± 6.26	69.738	<0.001^b^*
Education (years)	14.94 ± 2.69	12.89 ± 3.84	15.15 ± 1.95	12.84 ± 3.54	3.169	0.029^c^*
Duration of illness (months)	41.36 ± 15.67	47.05 ± 23.09	NA	NA	−0.888	0.381^d^
HAMD-17 score	22.57 ± 3.09	23.10 ± 3.60	NA	NA	−0.483	0.632^d^

^a^The *P*-values of gender distribution among the three groups were obtained using the chi-square test. *Post hoc t*-test: *P* = 1 (Younger TRD vs. Younger HCs), *P* = 1 (Older TRD vs. Older HCs).

^b^*P*-value from one-way analysis of variance tests. *Post hoc t*-test: *P* = 0.336 (Younger TRD vs. Younger HCs), *P* = 0.907 (Older TRD vs. Older HCs).

^c^*P*-value from one-way analysis of variance tests. *Post hoc t*-test: *P* = 0.835 (Younger TRD vs. Younger HCs), *P* = 0.958 (Older TRD vs. Older HCs).

^d^*P*-value from a two-sample *t*-test.

*Significant difference.

### Abnormal amplitude of low frequency fluctuations among four groups

**FIGURE 1:**
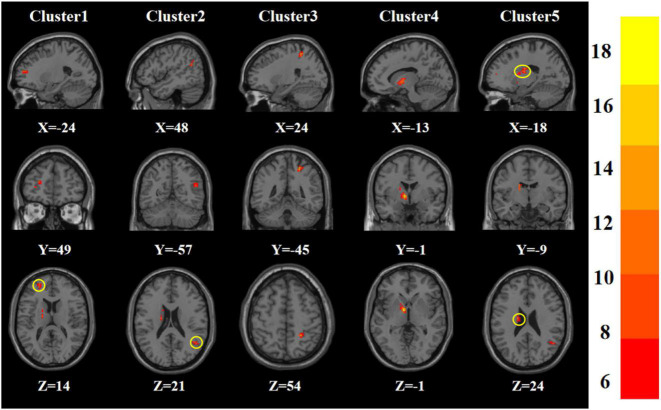
Statistical maps showing ANOVA result of ALFF abnormalities among patients with younger TRD, older TRD group, younger HC group, and older HC group (GRF corrected). The color bars indicate the *F*-value.

Age, gender, years of education, and frame displacement were used as covariates. One-way ANOVA revealed statistically significant differences in ALFF among the four groups in the left middle frontal gyrus, right middle temporal gyrus, right postcentral gyrus, left pallidum, and left caudate nucleus (Table 2 and Figure 1).

**TABLE 2:** Brain areas with different ALFF signals for four groups.

Clusters	Brain regions	Peak coordinates (MNI)			Cluster size	*F*-values
				
		X	Y	Z		
1	Left middle frontal gyrus	−24	49	14	31	8.415
2	Right middle temporal gyrus	48	−57	21	22	9.761
3	Right postcentral gyrus	24	−45	54	25	14.442
4	Left pallidum	−13	−1	−1	55	10.340
5	Left caudate nucleus	−18	−9	24	23	10.413

One-way ANOVA, P < 0.005, GRF corrected, cluster size > 20.

### Abnormal amplitude of low frequency fluctuations in younger treatment-resistant depression group vs. older treatment-resistant depression group

**FIGURE 2:**
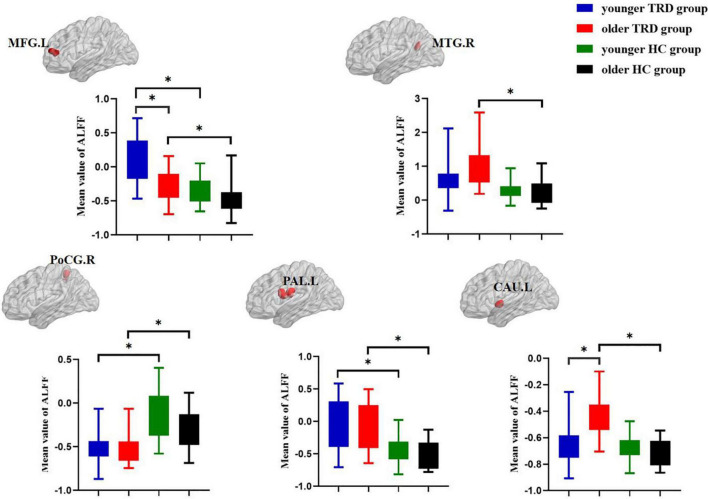
*Post hoc* two-sample *t*-tests (Bonferroni corrected) comparison showing ALFF values differences at peak voxel between each pair group (younger TRD group vs. older TRD group, younger TRD group vs. younger HC group, older TRD group vs. older HC group, younger HC group vs. older HC group). MFG.L, Left middle frontal gyrus; MTG.R, Right middle temporal gyrus; PoCG.R, Right postcentral gyrus; PAL.L, Left pallidum; CAU.L, Left caudate nucleus. *P < 0.0125.

Compared to the older TRD group, the younger TRD group had increased ALFF in the left middle frontal gyrus and decreased ALFF in the left caudate nucleus (Figure 2).

### Abnormal amplitude of low frequency fluctuations in younger treatment-resistant depression group vs. younger healthy control group

Compared with the younger HC group, the younger TRD group had increased ALFF in the left middle frontal gyrus and left pallidum, and decreased ALFF in the right postcentral gyrus (Figure 2).

### Abnormal amplitude of low frequency fluctuations in older treatment-resistant depression group vs. older healthy control group

Compared with the older HC group, the older TRD group had increased ALFF in the left middle frontal gyrus, right middle temporal gyrus, left pallidum, and left caudate nucleus, and decreased ALFF in the right postcentral gyrus (Figure 2).

### Abnormal amplitude of low frequency fluctuations in younger healthy control group vs. older healthy control group

There was no significant difference in the comparison of ALFF between the younger HC group and the older HC group (Figure 2).

### Correlation between amplitude of low frequency fluctuations and clinical symptoms

**FIGURE 3:**
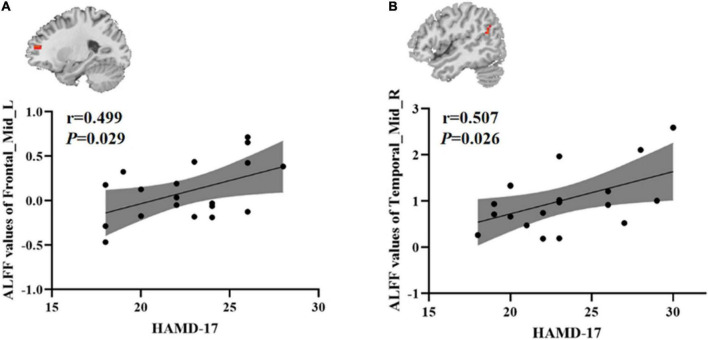
Positive correlation between the ALFF values of abnormal brain regions and the HAMD-17 scores: **(A)** ALFF values in the younger TRD group; **(B)** ALFF values in the older TRD group; Frontal_Mid_L, Left middle frontal gyrus; Temporal_Mid_R, Right middle temporal gyrus; ALFF, amplitude of low-frequency fluctuations; HAMD-17, 17-item Hamilton Rating Scale for Depression.

To test whether there was a correlation between clinical characteristics and abnormal brain regions ALFF in the younger TRD group and the older TRD group, we further performed a Pearson correlation analysis. We found that the left middle frontal gyrus ALFF values in the younger TRD group were positively correlated with HAMD-17 scores (*r* = 0.499, *P* = 0.029). In addition, the right middle temporal gyrus ALFF values in the older TRD group were positively correlated with HAMD-17 scores (*r* = 0.507, *P* = 0.026) (Figure 3).

## Discussion

To our knowledge, this is the first study using the ALFF method to analyze abnormalities in the physiopathological mechanisms of the brain between younger TRD and older TRD. The present study found no significant differences in clinical symptoms between younger and older TRD, but abnormal neuronal functional activity in some brain regions, with abnormalities associated with cognitive control networks (CCN) and reward networks. Compared to the matched HC group, TRD also exhibited abnormalities in some brain regions at different ages. The older TRD showed more extensive ALFF abnormalities than younger TRD. This study provides new insights into the differences in physiopathological mechanisms in patients with TRD at different ages.

This study found that the younger TRD group had increased ALFF in the left middle frontal gyrus than the older. The middle frontal gyrus is an important component of the dorsolateral prefrontal cortex (DLPFC) and an important component of the CCN, which is closely associated with negative emotions, top-down attention, and working memory (Fales et al., 2009; Wang et al., 2016; Egorova et al., 2018). Patients with MDD with DLPFC damage tend to show low interest in things, memory loss, and lack of motivation (Hamilton et al., 2012; Kang et al., 2012; Martin et al., 2017). Previous studies have found that EOD has increased ALFF in the superior frontal gyrus than LOD, suggesting that hyperactivity of the superior frontal gyrus in the resting state may provoke strong negative affect for the individual (Guo et al., 2012). Another study also showed that regional homogeneity (ReHo) was increased in the right inferior frontal triangular gyrus of the EOD than in the LOD, suggesting that abnormal functional activity in the prefrontal lobe helps to distinguish the EOD from the LOD (Zhang et al., 2021b). Therefore, the results of the present study suggest that the hyperactivity of the left middle frontal gyrus in the resting state in younger TRD may be related to the high level of stress caused by the life and work environment of young people. In addition, we further found that ALFF in the left middle frontal gyrus of the younger TRD group was positively correlated with HAMD-17 scores, whereas this was not found in the older TRD. This suggests that the left middle frontal gyrus may be a neuroimaging marker for young TRD and is an important brain region for distinguishing younger TRD from older TRD.

We found that ALFF was decreased in the left caudate nucleus in the younger TRD group compared to the older TRD group. The caudate nucleus is an important component of the striatum and is one of the central nodes of emotional processing (Pizzagalli et al., 2009; Stoy et al., 2012). The caudate nucleus is involved in the cortico-striato-pallidum-thalamus emotion regulation loop, which regulates the body’s response to external stimuli and maintains the balance of emotion regulation (Alexander et al., 1990; Haber and Calzavara, 2009; Peters et al., 2016). Meanwhile, the caudate nucleus is also an important component of the reward network and is involved in pleasure deficit and motivated reward processing in humans (Macpherson and Hikida, 2019; Cao et al., 2021). Previous studies found that ALFF in the right caudate nucleus was significantly increased in the MDD group than in the HC group, suggesting that abnormal spontaneous brain activity in the caudate nucleus may be associated with MDD (Liu et al., 2014; Chen et al., 2022). Another study also found that ketamine improved patients’ emotional perception through its modulatory effect on the caudate nucleus in TRD patients (Murrough et al., 2015). Therefore, the results of this study suggest that the degree of functional impairment of the left caudate nucleus is more severe in older TRD patients than in younger TRD. This further suggests that different physiopathological mechanisms may exist in patients with TRD at different ages of onset.

We found increased ALFF in the right middle temporal gyrus and left caudate nucleus in the older TRD group compared to the older HC group, which was not found in the younger TRD group compared to the younger HC group. Middle temporal gyrus is involved in emotional perception, audiovisual processing, memory and social cognitive functions, and is also an important component of the default mode network (DMN) (Raichle et al., 2001; Raichle and Snyder, 2007; Xu et al., 2019; Liu M. et al., 2021). Several previous studies have shown abnormalities in the functional activity of the DMN in patients with TRD, and the DMN varies by disease stage and age (de Kwaasteniet et al., 2015; Huang et al., 2020; Woody et al., 2021). A work showed that early-onset recurrent depression was increased ReHo in the right middle temporal gyrus than in the younger HC group, suggesting that this partially compensatory elevation of DMN may be one of the causes of abnormal brain function in early-onset recurrent depression (Sun et al., 2022). Therefore, the results of this study suggest that dysfunction and abnormalities of the right middle temporal gyrus and left caudate nucleus in older patients with TRD may be an important pathogenetic mechanism in younger patients with TRD. In addition, we found that ALFF values in the right middle temporal gyrus of the older TRD group were positively correlated with HAMD-17 scores, suggesting that this region may be an important neuroimaging marker and potential therapeutic target for older TRD patients. However, whether ALFF in this brain region associated with clinical symptoms can be a valid marker of TRD progression needs to be further elucidated.

Interestingly, compared with the two matched HC groups, the two subtype TRD groups had increased ALFF in the left middle frontal gyrus, left pallidum, and decreased ALFF in the right postcentral gyrus. The pallidum is not only a component of the striatum, but also a transmission node connecting the prefrontal cortex to the amygdala, which is closely associated with motivation and reward circuits in MDD patients (Smith et al., 2009; Knowland et al., 2017). Previous studies have found that the functional connectivity (FC) of median cingulate and paracingulate gyri and left pallidum was decreased in MDD patients compared to the HC group (Huang et al., 2021). In addition, a review also showed that vagus nerve stimulation and deep brain stimulation can reverse striatal abnormalities and thus alleviate TRD symptoms (Mohr et al., 2011). The postcentral gyrus belongs to the somatosensory-motor area, which is a higher-level center for the regulation of somatosensory and motor functions in the human body and an important part of the frontoparietal network, and is closely related to executive control and emotion management functions (Zhang et al., 2019; Liu M. et al., 2021). The postcentral gyrus plays an important role in the physiopathological mechanisms of TRD, and abnormalities of the postcentral gyrus predispose therefore TRD patients to somatic disorders (Klok et al., 2019). Previous studies have also found that electroconvulsive therapy can alleviate residual dysfunction in depression by reversing abnormal FC in the middle occipital gyrus and postcentral gyrus. Therefore, the results of this study suggest that cognitive control, reward motivation, and somatosensory-motor function were impaired in patients with TRD at different ages, independent of age of onset.

Some limitations need to be noted. First, for ethical reasons, TRD patients were not discontinued from antidepressants prior to enrollment. Therefore, we do not exclude the potential effects of antidepressants on brain function. Second, we only compared the differences in brain function between the TRD and HC groups, but the nTRD group was not included in this study. Therefore, the results of brain regions with abnormal ALFF (TRD group vs. HC group) only suggest an association with major depression and lack the specificity of TRD pathophysiology, which needs further study in the future. Third, only one clustering method was used to analyze the images in this study, and we will use different clustering methods to compare the results in future studies to improve the scientific significance of the results of this study. Finally, the small sample size of the present study limited the age classification range of the subjects. Therefore, we will further expand the sample size in future studies to improve the scientific value of this study.

## Conclusion

To summarize, we found that different neuropathological mechanisms may exist in TRD patients of different ages, especially in the left middle frontal gyrus and left caudate nucleus. This study is beneficial to provide potential key targets for clinical treatment of TRD patients in different age groups.

## Data availability statement

The raw data supporting the conclusions of this article will be made available by the authors, without undue reservation.

## Ethics statement

The experimental protocol was approved by the Ethics Committee of Guang’anmen Hospital, China Academy of Chinese Medical Science (NO. 2017-021-SQ), Trial registration, China Clinical Trials Registry, chiCTR1800014277. All patients signed an informed consent form before enrollment. The patients/participants provided their written informed consent to participate in this study. Written informed consent was obtained from the individual(s) for the publication of any potentially identifiable images or data included in this article.

## Author contributions

JF conceived and designed the experiments and revised the manuscript. JS collected cases, analyzed data, and wrote manuscript. CG analyzed data and revised manuscript. YM, ZD, ZW, YiL, LC, DG, XL, and KX collected cases and analyzed data. YH scanned the subjects. XY and XX evaluated patients and collected cases. YoL revised the manuscript. All authors contributed to the article and approved the submitted version.

## Footnotes

1
http://www.rfmri.org/DPARSF
